# Supplementary material for: Antifungal therapy in the management of fungal secondary infections in COVID-19 patients: A systematic review and meta-analysis
Source: PLoS One. 2022 Jul 28;17(7):e0271795. doi: 10.1371/journal.pone.0271795 (PMC9333218; doi:10.1371/journal.pone.0271795)
Supplement: S1 Appendix — (DOCX) [file pone.0271795.s002.docx]

**Supplementary Appendix S1: Details of PICOS format for study inclusion criteria**

| **PICOS format** | |
| --- | --- |
| **Participants** | - COVID-19 patients with fungal secondary infections - Either gender of any ages - At any clinical stages of diseases with or without other comorbidities |
| **Intervention** | - Use of antifungal agent either as mono- or as combination therapy |
| **Comparators** | - Intervention group (any AFT therapy) compared with placebo or standard care, if studies available (no any such studies identified) - For cohort studies (Prospective and retrospective studies), comparison was performed within studies in term of primary outcomes (all-cause mortality) among patient using AFT and patients without AFT, mono or combination antifungal therapy (AFT) and duration of AFT (≤28 days or >28 days) for fungal secondary infections among COVID-19 patients |
| **Outcomes** | - Prevalence of fungal secondary infections in COVID-19 patients - All-cause mortality among COVID-19 patients with fungal secondary infections using AFT and patients without AFT - All-cause mortality from fungal secondary infections among COVID-19 patient using mono or combination antifungal therapy (AFT) - All-cause mortality associated with the duration of AFT (≤28 days or >28 days) used in the management of fungal secondary infections among COVID-19 patients |
| **Study designs** | Case reports, case series, prospective studies, retrospective studies and clinical trails |
