# Supplementary material for: Antifungal therapy in the management of fungal secondary infections in COVID-19 patients: A systematic review and meta-analysis
Source: PLoS One. 2022 Jul 28;17(7):e0271795. doi: 10.1371/journal.pone.0271795 (PMC9333218; doi:10.1371/journal.pone.0271795)
Supplement: S2 Appendix — (DOCX) [file pone.0271795.s003.docx]

**Supplementary Appendix S2: Details on search strategies applied in various databases**

| **Database** | **Search details** | **Results** |
| --- | --- | --- |
| **PubMed** | ((antifungal therapy [Text Word] OR antifungal agents [MeSH Terms] OR voriconazole [MeSH Terms] OR isavuconazole [Text Word] OR liposomal amphotericin B [Text Word] OR amphotericin B [MeSH Terms] OR fluconazole [MeSH Terms] OR azoles [MeSH Terms]) AND (fungal infections [Text Word] OR mycoses [MeSH Terms] OR mucormycosis [MeSH Terms] OR mucor [MeSH Terms] OR black fungus [Text Word] OR zygomycosis [MeSH Terms] OR yellow fungus [Text Word] OR white fungus [Text Word] OR aspergillosis [MeSH Terms] OR aspergillus [MeSH Terms] OR aspergillus fumigatus [MeSH Terms] OR aspergillus flavus [MeSH Terms] OR azole resistant aspergillus [Text Word] OR candida [MeSH Terms] OR candida auris [Text Word] OR histoplasmosis [MeSH Terms] OR histoplasma [MeSH Terms] OR Cryptococcus [MeSH Terms] OR blastomycosis [MeSH Terms] OR coccidioidomycosis [MeSH Terms] OR candidemia [MeSH Terms] OR saccharomyces [MeSH Terms] OR pneumonia, pneumocystis [MeSH Terms] OR pneumocystis [MeSH Terms] OR pneumocystis[Text Word] OR candidiasis[MeSH Terms])) AND (COVID-19 [MeSH Terms] OR 2019 novel coronavirus infection [Text Word] OR coronavirus disease 2019 [Text Word] OR 2019- nCov infection [Text Word] OR severe acute respiratory syndrome coronavirus-2 [Text Word] OR SARS2 [Text Word] OR SARS-CoV-2 [MeSH Terms] OR 2019- nCoV[Text Word])    **Filters applied:**  Included documents type during search: Books and documents, Clinical trial, Meta-analysis, Randomized control trail, Review, Systematic review, English, Humans  Publications Date: 2020 to 2021 | **54** |
| **Scopus** | TITLE-ABS-KEY (antifungal therapy) OR TITLE-ABS-KEY (antifungal agents) OR TITLE-ABS-KEY (voriconazole) OR TITLE-ABS-KEY (isavuconazole) OR TITLE-ABS-KEY (liposomal amphotericin B) OR TITLE-ABS-KEY (amphotericin B) OR TITLE-ABS-KEY (fluconazole) OR TITLE-ABS-KEY (azoles) AND TITLE-ABS-KEY (fungal infections) OR TITLE-ABS-KEY (mycoses) OR TITLE-ABS-KEY (mucormycosis) OR TITLE-ABS-KEY (mucor) OR TITLE-ABS-KEY (black fungus) OR TITLE-ABS-KEY (zygomycosis) OR TITLE-ABS-KEY (yellow fungus) OR TITLE-ABS-KEY (white fungus) OR TITLE-ABS-KEY (aspergillosis) OR TITLE-ABS-KEY (aspergillus) OR TITLE-ABS-KEY (aspergillus fumigatus) OR TITLE-ABS-KEY (aspergillus flavus) OR TITLE-ABS-KEY (azole resistant aspergillus) OR TITLE-ABS-KEY (candida) OR TITLE-ABS-KEY (candida auris) OR TITLE-ABS-KEY (histoplasmosis) OR TITLE-ABS-KEY (histoplasma) OR TITLE-ABS-KEY (Cryptococcus) OR TITLE-ABS-KEY (blastomycosis) OR TITLE-ABS-KEY (coccidioidomycosis) OR TITLE-ABS-KEY (candidemia) OR TITLE-ABS-KEY (saccharomyces) OR TITLE-ABS-KEY (pneumocystis pneumonia) OR TITLE-ABS-KEY (pneumocystis) OR TITLE-ABS-KEY (pneumocystis) OR TITLE-ABS-KEY (candidiasis) AND TITLE-ABS-KEY (COVID-19) OR TITLE-ABS-KEY (2019 novel coronavirus infection) OR TITLE-ABS-KEY ( coronavirus disease 2019) OR TITLE-ABS-KEY (2019 nCov infection) OR TITLE-ABS-KEY (severe acute respiratory syndrome coronavirus-2) OR TITLE-ABS-KEY (SARS2) OR TITLE-ABS-KEY (SARS CoV2) OR TITLE-ABS-KEY (2019 nCoV))  **Filters applied:**  Search field: TITLE-ABS-KEY  Language: English  Documents Type: Articles  Publication Date: 2020-2021 | **172** |
| **Web of Science of core collection** | (TS=(antifungal therapy) OR TS=(antifungal agents) OR TS= (voriconazole) OR TS= (isavuconazole) OR TS=(liposomal amphotericin B) OR TS=(amphotericin B) OR TS=(fluconazole) OR TS=(azoles) AND TS=(fungal infections) OR TS=(mycoses) OR TS= (mucormycosis) OR TS=(mucor) OR TS=(black fungus) OR TS=(zygomycosis) OR TS=(yellow fungus) OR TS=(white fungus) OR TS=(aspergillosis) OR TS=(aspergillus) OR TS=(aspergillus fumigatus) OR TS=(aspergillus flavus) OR TS=(azole resistant aspergillus) OR TS=(candida) OR TS=(candida auris) OR TS=(histoplasmosis) OR TS=(histoplasma) OR TS=(Cryptococcus) OR TS=(blastomycosis) OR TS=(coccidioidomycosis) OR TS=(candidemia) OR TS=(saccharomyces) OR TS=(pneumocystis pneumonia) OR TS=(pneumocystis) OR TS=(pneumocystis) OR TS=(candidiasis) AND TS=(COVID-19) OR TS=(2019 novel coronavirus infection) OR TS=( coronavirus disease 2019) OR TS=(2019 nCov infection) OR TS=(severe acute respiratory syndrome coronavirus-2) OR TS=(SARS2) OR TS=(SARS CoV2) OR TS=(2019 nCoV))  **Filters applied:**  Search field: Title  Timespan:January-1-2020 to June-30-2021  Document searched type: Article, Abstracts of the published item, Meeting abstracts, Meeting summary | **103** |
| **Cochrane Library Central** | (antifungal therapy) OR (antifungal agents) OR (voriconazole) OR (isavuconazole) OR (liposomal amphotericin B) OR (amphotericin B) OR (fluconazole) OR (azoles) AND (fungal infections) OR (mycoses) OR (mucormycosis) OR (mucor) OR (black fungus) OR (zygomycosis) OR (yellow fungus) OR (white fungus) OR (aspergillosis) OR (aspergillus) OR (aspergillus fumigatus) OR (aspergillus flavus) OR (azole resistant aspergillus) OR (candida) OR (candida auris) OR (histoplasmosis) OR (histoplasma) OR (Cryptococcus) OR (blastomycosis) OR (coccidioidomycosis) OR (candidemia) OR (saccharomyces) OR (pneumocystis pneumonia) OR (pneumocystis) OR (pneumocystis) OR (candidiasis) AND (COVID-19) OR (2019 novel coronavirus infection) OR ( coronavirus disease 2019) OR (2019 nCov infection) OR (severe acute respiratory syndrome coronavirus-2) OR (SARS2) OR (SARS CoV2) OR (2019 nCoV) in Title Abstract Keyword    **Filters applied:**  Search field: Title Abstract Keyword  Document type: Trials (Word variations have been searched)  Publication years: Jan 2020 and June 2021 | **6** |
| **MedrRxiv & bioRxiv** | Above mentioned serach terms were adopted and sutiabley apllied to amended necessary documents | **38** |
| **Google Scholar** | Above mentioned serach terms were adopted and sutiabley apllied to amended necessary documents | **20** |
