# Supplementary material for: Antifungal therapy in the management of fungal secondary infections in COVID-19 patients: A systematic review and meta-analysis
Source: PLoS One. 2022 Jul 28;17(7):e0271795. doi: 10.1371/journal.pone.0271795 (PMC9333218; doi:10.1371/journal.pone.0271795)
Supplement: S1 Table — (DOCX) [file pone.0271795.s004.docx]

**Supplementary Table S1: Details of excluded literatures form the review**

| **S.N** | **First author, country, Year** | **Title of study** | **Reason for exclusion** |
| --- | --- | --- | --- |
|  | Alanio A et al, France  2020 | Prevalence of putative invasive pulmonary aspergillosis in critically ill patients with COVID-19 | Correspondence |
|  | Jain A et al, (not available), 2020 | A recovered case of COVID-19 myocarditis treated with IV immunoglobulin | Conference proceeding |
|  | Warris A et al, UK & Canada, 2021 | Covid-19 associated pulmonary aspergillosis | Editorial |
|  | Oliva A et al, Italy, 2020 | Co‐infection of SARS‐CoV‐2 with Chlamydia or Mycoplasma pneumoniae | Original article that did not use antifungal agents |
|  | Borman A M et al, UK, 2020 | COVID-19-Associated Invasive Aspergillosis: | Registry data  (Description about testing) |
|  | Chowdharya A, India, 2020 | The lurking scourge of multidrug resistant Candida auris in times of  COVID-19 pandemic | Short communication |
|  | Santosh ABR, India, 2021 | Fungal infections of oral cavity: diagnosis, management,  and association with COVID-19 | Review |
|  | Henry A et al, UK, | Welcome to the 16th volume of future microbiology | Foreword |
|  | Dare BL et al, France, 2021 | Toward the personalized and integrative management of voriconazole dosing during COVID-19-associated pulmonary aspergillosis | Research letter |
|  | Garcia BM et al, USA, 2021 | Disseminated-cutaneous Sporotrichosis in an immunocompetent adult | Case report (COVID 19 negative) |
|  | Sarrazyn C et al, Belgium, 2021 | Incidence, risk factors, timing, and outcome of influenza versus  COVID-19–associated putative invasive aspergillosis | Editorial |
|  | Shadrach BJ et al, India, 2021 | Invasive pulmonary aspergillosis in a COVID-19 recovered patient:  unravelling an infective sequalae of the SARS-CoV-2 virus | Editorial |
|  | Poignon C et al, France,  2020 | Invasive pulmonary fusariosis in an immunocompetent critically ill  patient with severe COVID-19 | Editorial |
|  | Allaw F et al, Lebanon, 2020 | First Candida auris Outbreak during a COVID-19 Pandemic in a  Tertiary-Care Center in Lebanon | Half of the study subjects were having non-COVID related fungal co-infections |
|  | Feldman C et al, South Africa, 2021 | The role of co-infections and secondary infections in patients with COVID-19 | Review |
|  | Dupont D et al, France, 2021 | Pulmonary aspergillosis in critically ill patients with Coronavirus Disease 2019 (COVID-19) | Brief report |
|  | Lai CC et al, Taiwan, 2021 | COVID-19 associated with pulmonary aspergillosis | Review article |
|  | Contou D et al, France, 2020 | Bacterial and viral co-infections in patients with severe SARS-CoV-2 pneumonia admitted to a French ICU | Original article that did not use antifungal agents |
|  | Savil D et al, Italy, 2020 | Uncommon presentation of allergic bronchopulmonary aspergillosis  during the COVID-19 lockdown: a case report | Case report of non-fungal co-infections |
|  | Armstrong-James D et al, UK, 2020 | Confronting and mitigating the risk of COVID-19 associated pulmonary  aspergillosis | Editorial |
|  | Rubin ES et al, USA, 2020 | Detection of COVID-19 in a Vulvar Lesion | Short communication |
|  | Villanueva-Lozano H et al, Mexico, 2021 | Outbreak of Candida auris infection in a COVID-19 hospital in Mexico | Editorial |
|  | Lamoth F et al, Switzerland  2020 | Incidence of invasive pulmonary aspergillosis among critically ill  COVID-19 patients | Editorial |
|  | Wu1 F et al, China, 2020 | Clinical characteristics of COVID-19 infection in chronic obstructive pulmonary disease: a multicenter, retrospective, observational study | Retrospective,  Observational study  of non-fungal co-infections |
|  | Veerdonk, FLVD et al, Netherlands, 2020 | Invasive Aspergillus Tracheobronchitis emerging as a highly lethal  complication of severe Influenza | Article on non-fungal co-infections |
|  | Lescure FX et al, France,  2020 | Clinical and virological data of the first cases of COVID-19 in Europe | A case series of non-fungal co-infections |
|  | Gouziena L et al, France, 2021 | Invasive Aspergillosis associated with Covid-19 | Short communication |
|  | Bhatt K et al, USA, 2021 | High mortality co-infections of COVID-19 patients: mucormycosis and other fungal infections | Review article |
|  | Wang J et al, China, 2020 | Clinical characteristics of invasive pulmonary aspergillosis in patients with  COVID-19 in Zhejiang, China | Research letter |
|  | Silva LN et al, Brazil, 2020 | Fungal Infections in COVID-19-positive patients: a lack of optimal  treatment options | Narrative review |
|  | Marr KA et al, USA, 2021 | Aspergillosis Complicating Severe Coronavirus Disease | Synopsis |
|  | Helleberg M et al, Denmark, 2020 | Invasive aspergillosis in patients with severe COVID-19 pneumonia | Editorial |
|  | Rutsaert L et al, Belgium, 2020 | COVID-19-associated invasive pulmonary aspergillosis | Editorial |
|  | Spadea M et al, Italy, 2020 | Successfully treated severe COVID-19 and invasive aspergillosis in early hematopoietic cell transplantation setting | Editorial |
|  | Liesenborghs L et al, Belgium, 2021 | Itraconazole for COVID-19: preclinical studies and a proof-of-concept  randomized clinical trial | Research paper |
|  | Blaize, M et al, France, 2020 | Fatal Invasive Aspergillosis and Coronavirus Disease in an Immunocompetent Patient | Research letter |
|  | Hoenigi M et al, USA, 2020 | Invasive fungal disease complicating Coronavirus Disease 2019: When It Rains, It Spores | Editorial commentary |
|  | Clementea MG et al, Espana, 2021 | Can SARS-CoV-2 be a Risk Factor for Pulmonary Aspergillosis? | Editorial |
|  | Almeida JND, Brazil, 2021 | Emergence of Candida auris in Brazil in a COVID-19 Intensive Care Unit | Brief report |
|  | Nestler BSM et al, USA, 2021 | Fungal superinfection in patients with COVID-19: Role of antifungal  stewardship? | Commentary |
|  | Gonzalez-Garcia M et al, Denmark, Cuba, Brazil Germany, 2021 | Antimicrobial Activity of Cyclic-Monomeric and Dimeric  Derivatives of the Snail-Derived Peptide Cm-p5 against Viral  and Multidrug-Resistant Bacterial Strains | Testing techniques |
|  | Koehler P, Canada, 2020 | Defining and managing COVID-19-associated pulmonary aspergillosis: the 2020 ECMM/ISHAM consensus criteria for research and clinical guidance | Review |
|  | Sasoni N et al, Argentina, 2021 | SARS-CoV-2 and Aspergillus section Fumigati coinfection in an immunocompetent patient treated with corticosteroids | Note |
|  | Verweij PE et al, Netherlands, 2020 | Review of infuenza-associated pulmonary aspergillosis in ICU patients and proposal for a case defnition: an expert opinion | Conference reports and expert panel |
|  | Ichai P et al, France, 2020 | Impact of negative air pressure in ICU rooms on the risk of pulmonary  aspergillosis in COVID-19 patients | Research letter |
|  | Ahmed N et al, Egypt, 2021 | Invasive Fungal Sinusitis in Post COVID-19 Patients: A New Clinical Entity | Editorial |
|  | Nori P et al, USA, 2020 | Bacterial and fungal coinfections in COVID-19 patients hospitalized  during the New York City pandemic surge | Concise communication |
|  | De Macedo PM et al, Brazil, 2020 | COVID-19 and acute juvenile paracoccidioidomycosis coinfection | Symposium |
|  | Leon R et al, Spain, 2021 | Bacterial and Fungal Infections in Critically Ill Patients with Covid-19 Pneumonia: An Observational Study | Short communication |
|  | Basso RP et al, Brazil, 2020 | COVID-19-Associated Histoplasmosis in an AIDS Patient | Short communication |
|  | Sarkar,S et al, India, 2021 | COVID‐19 and orbital mucormycosis | Editorial |
|  | Lipner SR et al, USA, 2020 | Recommendations for Diagnosis and Treatment of Onychomycosis During the COVID-19 Pandemic | Editorial |
|  | Wu S et al, China, 2020 | Dynamic Immune Response Profiles and Recovery of a COVID-19 Patient with Coinfection of Aspergillus fumigatus and Other Baseline Diseases: A Case Report | Editorial |
|  | Solares CA et al, Georgia, 2021 | Interdisciplinary Teamwork within the Medical Profession: The Way of the Future | Editorial |
|  | Cai1 S et al, China, 2020 | A complex COVID-19 case with rheumatoid arthritis treated with tocilizumab | Case based review |
|  | Antinori S et al, Italy, 2020 | Bacterial and fungal infections among patients with SARS-CoV-2 pneumonia | Review |
|  | Antinoria S et al, Italy, 2020 | Invasive pulmonary aspergillosis complicating SARS-CoV-2 pneumonia: A  diagnostic challenge | Commentary |
|  | Lahmer T et al, Germany, 2020 | Invasive pulmonary aspergillosis in severe coronavirus disease 2019  pneumonia | Editorial |
|  | Rawson TM et al, UK, 2021 | Understanding the role of bacterial and fungal infection in COVID-19 | Commentary |
|  | Di Pilato V et al, Italy, 2021 | Molecular Epidemiological Investigation of a Nosocomial Cluster of C. auris: Evidence of Recent Emergence in Italy and Ease of Transmission during the COVID-19 Pandemic | Diagnostic |
|  | Chaudhry Z et al, UK, 2021 | Short durations of corticosteroids for hospitalised COVID-19 patients are associated with a high readmission rate | Editorial |
|  | Meijer EFJ et al, Netherland & Brazil, 2020 | Azole-Resistant COVID-19-Associated Pulmonary Aspergillosis in an Immunocompetent Host: A Case Report | Case report  (Duplicate-same data presented in case series) |
|  | Zeng JH et al, China, 2020 | First case of COVID‑19 complicated with fulminant myocarditis: a case  report and insights | Case report  (antifungal agents not used) |
|  | Karaba SM et al, USA, 2020 | Prevalence of co-infection at the time of hospital admission in COVID-19 Patients, A multicenter study | Major research  on non-fungal co-infections |
|  | Vidal CG et al, Spain, 2021 | Incidence of co-infections and superinfections in hospitalized patients  with COVID-19: a retrospective cohort study | Original article that did not use antifungal agents |
|  | Dellieere S et al, France, 2021 | Risk factors associated with COVID-19-associated pulmonary  aspergillosis in ICU patients: a French multicentric retrospective  cohort | Original article that did not use antifungal agents |
|  | Yang X et al, China, 2020 | Clinical course and outcomes of critically ill patients withnSARS-CoV-2 pneumonia in Wuhan, China: a single-centered, retrospective, observational study | Original article that did not use antifungal agents |
|  | Vijay S et al, India, 2021 | Secondary Infections in Hospitalized COVID-19 Patients: Indian Experience | Original article that did not use antifungal agents |
|  | Maes M et al, UK, 2020 | Ventilator‑associated pneumonia in critically ill patients with COVID‑19 | Original article that did not use antifungal agents |
|  | Lv Z et al, China, 2020 | Clinical characteristics and co-infections of 354 hospitalized patients  with COVID-19 in Wuhan, China: a retrospective cohort study | Original article that did not use antifungal agents |
|  | Ferreira TG et al, France, 2021 | Recovery of a triazole-resistant Aspergillus fumigatus in respiratory specimen of COVID-19 patient in ICU – A case report | Case Report that did not involve antifungal agents |
|  | Hughes S et al, UK, 2020 | Bacterial and fungal coinfection among hospitalized patients with  COVID-19: a retrospective cohort study in a UK secondary-care setting | Original article that did not use antifungal agents |
|  | Razazi K et al, France, 2020 | Risks of ventilator‑associated pneumonia and invasive pulmonary aspergillosis in patients with viral acute respiratory distress syndrome related or not to Coronavirus 19 disease | Original article that did not use antifungal agents |
|  | Kariyawasam RM et al, Canada, 2021 | COVID-19 Associated Pulmonary Aspergillosis: Systematic Review and Patient-Level Meta-analysis | Systematic Review and Meta-analysis |
|  | Musuuza JS et al, USA, 2021 | Prevalence and outcomes of co-infection and superinfection with SARS-CoV-2 and other pathogens: A systematic review and metaanalysis | Systematic Review and Meta-analysis |
|  | Pemana J et al, Spain, 2020 | Fungal co-infection in COVID-19 patients: Should we be concerned? | Narrative Review |
|  | Peng J et al, China, 2021 | Fungal co-infection in COVID-19 patients: evidence from a systematic review and meta-analysis | Systematic Review and Meta-analysis |
|  | Mitaka H et al, USA, 2021 | Incidence and mortality of COVID-19- associated pulmonary baspergillosis: A systematic review and meta-analysis | Systematic Review and Meta-analysis |
|  | Ravini SA et al, India, 2021 | Rise of the phoenix: Mucormycosis in COVID-19 times | Full-text not available |
|  | Srirampur A et al, India, 2020 | Dematiaceous Fungal Colonization of the Bandage Contact Lens in a Patient Lost to Follow-up During the COVID-19 Crisis | Full-text not available |
|  | Bhagali R et al, India, 2021 | Post COVID-19 opportunistic candida retinitis: A case report | Full-text not available |
|  | Hansen S et al, Canada, 2021 | Ruxolitinib as adjunctive therapy for secondary hemophagocytic lymphohistiocytosis: A case series | Full-text not available |
