# Supplementary material for: Antifungal therapy in the management of fungal secondary infections in COVID-19 patients: A systematic review and meta-analysis
Source: PLoS One. 2022 Jul 28;17(7):e0271795. doi: 10.1371/journal.pone.0271795 (PMC9333218; doi:10.1371/journal.pone.0271795)
Supplement: S2 Table — (DOCX) [file pone.0271795.s005.docx]

**Supplementary Table S2: Summary of fungal secondary infections, antifungal therapy (AFT) used, duration of AFT and outcomes COVID-19 patients**

| **First author, Country, Year** | **Number of COVID-19 diagnosed with fungal co-infections** | **Patient prescribed with AFT** | | | | | **Duration of AFT (days)** | | **Outcomes**  **(Patient with fungal co-infections)** | | |
| --- | --- | --- | --- | --- | --- | --- | --- | --- | --- | --- | --- |
|  |  | **Mono Therapy** | **Dual therapy** | **Triple therapy and Quadruple therapy** | **Unknown AFT** | **Patients without fungal therapy (NFT)** | **≤28** | **>28** | **Death** | **Alive** | **Unknown** |
| **Case Report:** | | | | | | | | | | | |
| Haglund A et al, Denmark, 2021^23^ | 1  (CAPA) | 1  (VOR) | NA | NA | NA | NA | NA | 1 | NA | 1 | NA |
| Hakamifard A et al, Iran, 2020[24] | 1  (CAPA) | NA | 1  (VOR+AMB) | NA | NA | NA | 1 | NA | 1 | NA | NA |
| Sharma A et al, Australia, 2021[25] | 1  (CAPA) | 1  (VOR) | NA | NA | NA | NA | 1 | NA | NA | 1 | NA |
| Witting C et al, USA, 2021[26] | 1  (CAPA) | NA | 1  (VOR+MIC) | NA | NA | NA | 1 | NA | NA | 1 | NA |
| Deana C et al, Italy, 2021[27] | 1  (CAPA) | 1  (AMB) | NA | NA | NA | NA | NA | 1 | NA | 1 | NA |
| Nasri E et al, Iran, 2020[28] | 1  (CAPA) | 1  (AMB) | NA | NA | NA | NA | 1 | NA | 1 | NA | NA |
| Mohamed A et al, Ireland, 2021[29] | 1  (CAPA) | 1  (AMB) | NA | NA | NA | NA | 1 | NA | 1 | NA | NA |
| Schein F et al. France, 2020[30] | 1  (CAPA) | 1  (VOR) | NA | NA | NA | NA | 1 | NA | 1 | NA | NA |
| Trujillo H et al. Spain, 2020[31] | 1  (CAPA) | NA | 1  (AMB+ISA) | NA | NA | NA | 1 | NA | NA | 1 | NA |
| Prattes J et al, USA, 2021[32] | 1  (CAPA) | 1  (VOR) | NA | NA | NA | NA | 1 | NA | 1 | NA | NA |
| Alobaid K et al, Kuwait, 2021[33]  (2 cases) | 1  (CAPA) | NA | 1  (CAS+VOR) | NA | NA | NA | 1 | NA | 1 | NA | NA |
|  | 1  (CAPA) | NA | 1  (ANI+AMB) | NA | NA | NA | 1 | NA | 1 | NA | NA |
| Trovato L et al, Italy, 2020[34] | 1  (CAPA) | 1  (VOR) | NA | NA | NA | NA | 1 | NA | 1 | NA | NA |
| Saccaro LF et al, Italy, 2020[35] | 1  (CAPA) | NA | 1  (ISA+MIC) | NA | NA | NA |  | 1 | NA | 1 | NA |
| Bilani N et al, USA, 2020[36] | 1  (Pseudofungi) | 1  (VOR) | NA | NA | NA | NA | NR | NR | NA | 1 | NA |
| Fernandez NB et al, Argentina, 2021[37] | 1  (CAPA) | NA | 1  (ANI+VOR) | NA | NA | NA | 1 |  | 1 |  | NA |
| Patti RK et al, USA, 2020[38] | 1  (CAPA) | 1  (VOR) | NA | NA | NA | NA | NR | NR | NA | 1 | NA |
| Kakamad FH et al, Iraq, 2021[39] | 1  (CAPA) | NA | NA | NA | 1 | NA | NR | NR | NA | 1 | NA |
| Abdalla S et al, Qatar, 2020[40]  (Reports of 2 cases) | 1  (CAPA) | NA | 1  (ANI+AMB) | NA | NA | NA | 1 | NA | 1 | NA | NA |
|  | 1  (CAPA) | 1  (VOR) | NA | NA | NA | NA | NA | 1 | 1 | NA | NA |
| Imoto M et al, Japan, 2021[41] | 1  (CAPA) | NA | 1  (MIC+VOR) | NA | NA | NA | 1 | NA | 1 | NA | NA |
| Iwanaga Y et al, Japan, 2021[42] | 1  (CAPA) | 1  (AMB) | NA | NA | NA | NA | 1 | NA | 1 | NA | NA |
| Maini A et al, India, 2021[43] | 1  (Sinoorbital CAM) | NA | 1  (AMB+FLU) | NA | NA | NA | NA | 2 | NA | 1 | NA |
| Khatri A et al, USA, 2021[44] | 1  (Cutaneous CAM) | NA | 1  (AMB+POS) | NA | NA | NA | NR | NR | 1 | NA | NA |
| Arana C et al, Spain, 2021[45]  (Reports of 2 cases) | 1  (Rhinosinusal CAM) | NA | 1  (AMB+ISA) | NA | NA | NA | NA | 1 | NA | 1 | NA |
|  | 1  (Musculoskeletal CAM) | NA | 1  (AMB+ISA) | NA | NA | NA | NA | 1 | NA | 1 | NA |
| Krishna DS et al, India, 2021[46]  (Reports of 2 cases) | 1  (Fungal osteomyelitis & zygoma) | NA | 1  (AMB+ITR) | NA | NA | NA | NA | 1 | NA | 1 | NA |
|  | 1  (CAM of right maxilla) | NA | 1  (AMB+POS) | NA | NA | NA | NA | 1 | NA | 1 | NA |
| Garg D et al, India, 2021[18] | 1  (Pulmonary CAM) | 1  (AMB) | NA | NA | NA | NA | NA | 1 | NA | 1 | NA |
| Junior ESM et al, Brazil, 2020[47] | 1  (Gastrointestinal CAM) | NA | NA | NA | 1 | NA | NR | NR | 1 |  | NA |
| Revannavar SM et al, India, 2021[48] | 1  (CAM) | 1  (AMB) | NA | NA | NA | NA | 1 | NA | NA | 1 | NA |
| Sari AP et al, Indonesia, 2021[49] | 1  (CAC) | 1  (MIC) | NA | NA | NA | NA | 1 | NA | NA | 1 | NA |
| Chang CC et al, USA, 2020[50] | 1  (Coccidioidomycosis) | 1  (FLU) | NA | NA | NA | NA | NR | NA | NA | 1 | NA |
| Ventoulis I et al, Greece, 2020[51]  (Reports of 2 cases) | 1  (S. cerevisiae) | NA | 1  (ANI+FLU) | NA | NA | NA | 1 | NA | NA | 1 | NA |
|  | 1  (S. cerevisiae) | NA | 1  (ANI+FLU) | NA | NA | NA | 1 | NA | NA | 1 | NA |
| Bertolini M et al, Argentina, 202[52] | 1  (Histoplasmosis) | NA | 1  (AMB+ITR) | NA | NA | NA | 1 | NA | NA | 1 | NA |
| Khatib MY et al, Qatar, 2020[53] | 1  (Cryptococcemia) | NA | NA | 1  (ANI+AMB+FLUC) | NA | NA | NA | 1 | 1 | NA | NA |
| Seitz T et al, Austria, 2020[54] | 1  (CAC) | 1  (CAS) | NA | NA | NA | NA | 1 | NA | NA | 1 | NA |
| **Case series:** | | | | | | | | | | | |
| Meijer EFJ et al, Netherlands, 2020[55] | 13  (CAPA) | 7  (VOR) | 2  (CAS+AMB:1, CAS+VOR:1) | 1  (CAS+VOR+AMB) | NA | NA | NR | NR | 6 | 7 | NA |
| Benedetti MF et al, Argentina, 2021[56] | 5  (CAPA) | 6  (VOR:4, AMB: 1, FLU:1) | NA | NA | NA | NA | NR | NR | 1 | 4 | NA |
| Flikweert AW et al,  Netherlands, 2020[57] | 7  (CAPA) | NA | 6  (VOR+ANI) | NA | NA | NA | NR | NR | 3 | 4 | NA |
| **Sub-total (Case report & series)** | **63** | **32** | **26** | **2** | **2** | **0** | **21** | **12** | **26** | **37** | **NA** |
| **Cohort study (observational & retrospective studies):** | | | | | | | | | | | |
| Rothe K et al, Germany, 2021[58] | 12  (CAPA-9; CAC-3) | 23  (ECH:5, VOR:4, FLU:6, AMB:8) | NA | NA | NA | NA | NR | NR | NA | NA | 12 |
| Sen M et al, India, 2021[59] | 6  (CAM) | 1  (AMB) | 4  (POS+AMB) | 1  (POS+AMB+VOR) | NA | NA | 2 | 4 | NA | 6 | NA |
| Chen N et al, China, 2020[60] | 4  (CAC) | NA | NA | NA | 15 | NA | NR | NR | NA | NA | 4 |
| Permpalung N et al, USA, 2021[61] | 39  (CAPA) | NA | NA | NA | 28 | 11 | NR | NR | 22 | 17 | NA |
| White PL et al, UK, 2020[62] | 17  (Yeast infection) | 9  (FLU:6, VOR:1, CAS:2) | 6  (CAS+AMB:1, CAS+FLU:2, CAS+VOR:1, FLU+VOR:1, FLU+AMB:1) | NA | NA | 2 | NR | NR | 8 | 9 | NA |
| Koehler P et al, Germany, 2020[63] | 5  (CAPA) | 3  (VOR:2, ISA1) | 2  (CAS+VOR) | NA | NA | NA | NR | NR | 3 | 2 | NA |
| Maes M et al, UK, 2021[64] | 3  (CAPA) | 3  (AMB) | NA | NA | NA | NA | NR | NR | 1 | 2 | NA |
| Nasir N et al, Pakistan, 2020[65] | 9  (CAPA) | 5  (VOR:3, AMB:2) | NA | NA | NA | 4 | 9 | NR | 4 | 5 | NA |
| Bishburg E et al, USA, 2020[66] | 8  (CAC) | 4  (FLU:3, CAS:1) | 4  (CAS+FLU) | NA | NA | NA | NR | NR | 3 | 5 | NA |
| Lahmer T et al, Germany, 2021[67] | 11  (CAPA) | 11  (VOR: 5, ISA:1, AMB:5) | NA | NA | NA | NA | 11 | NA | 4 | 7 | NA |
| Arastehfar A et al, Iran, 2021[68] | 7  (CAC) | 2  (FLU) | 5  (CAS+FLU) | NA | NA | NA | NR | NR | 6 | 1 | NA |
| Fekkar A et al, France, 2021[69] | 7  (CAPA-4; CAPA+CAM-2; CAPA+CAC-1) | NA | 4  (VOR+CAS:3, AMB+CAS:1) | 1  (VOR+CAS+AMB)  1  (VOR+AMB+CAS+  ISA) | NA | 1 | 5 | 1 | 4 | 3 | NA |
| Roman-Montes CM et al, Mexico, 2020[70] | 14  (CAPA) | 12  (VOR:10, ANI:2) | NA | NA | NA | 2 | 6 | 5 | 8 | 5 | 1 |
| Segrelles-Calvo G et al, Spain, 2020[71] | 7  (CAPA) | 5  (ITA:4, AMB:1) | NA | NA | NA | 2 | 3 | 2 | 5 | 2 | NA |
| Mitaka H et al, USA, 2020[72] | 4  (CAPA) | 4  (VOR:3, CAS:1) | NA | NA | NA | NA | 3 | 1 | 4 |  | NA |
| Salmanton-García J et al, Germany, 2021[73] | 158  (CAPA) | 187  (AMB:36, ANI:10, CAS:13, MIC:1, IBR:1, VOR:98, ISA:23, POS:4, FLU:1) | NA | NA | NA | NA | NR | NR | 119 | 39 | NA |
| Søgaard KK et al, Switzerland, 2021[74] | 3  (CAPA-2; CAC-1) | 7  (FLU:1, CAS:3, ANI:1, VOR:2) | NA | NA | NA | NA | NR | NR |  | NA | 3 |
| Versyck M et al, France, 2021[75] | 2  (CAPA) | 2  (VOR) | NA | NA | NA | NA | 2 |  | 2 | NA | NA |
| Salehi M et al, Iran, 2020[76] | 53  (CAC) | 35  (FLU:21, NYS:13, CAS:1) | 17  (FLU+NYS) | NA | NA | 1 | NR | NR | NA | NA | 53 |
| Buehler PK et al, Switzerland, 2020[77] | 34  (CAPA-5; CAC-29) | NA | NA | NA | 10 | NA | NR | NR | NA | NA | 34 |
| Seaton RA et al, UK, 2020[78] | 13  (Unknown) | 13  (CAS:7, FLU:5, VOR:1) | NA | NA | NA | NA | NR | NR | NA | NA | 13 |
| **Sub-total (Cohort studies)** | **416** | **326** | **42** | **3** | **53** | **23** | **41** | **13** | **193** | **103** | **120** |
| **Total** | **479** | **356** | **68** | **5** | **55** | **23** | **62** | **25** | **219** | **140** | **120** |
| *AFT: Antifungal therapy; AMB: Amphotericin B; ANI: Anidulafungin; BSAA: Broad spectrum antifungal agents; CAS: Caspofungin; CAC: COVID-19 Associated Candidemia; CAPA: COVID-19 Associated Pulmonary Aspergillosis; CAM: COVID-19 Associated Mucormycosis; ECH: Echinocandins; FLU: Fluconazole; FLUC: Flucytosine; IBR: Ibrexafungin; ISA: Isavuconazole; ITR: Itraconazole; MIC: Micafungin; NA: Not applicable; NR: Not reported; NYS: Nystatin; POS: Posaconazole; VOR: Voriconazole.* | | | | | | | | | | | |
