# Supplementary material for: Antifungal therapy in the management of fungal secondary infections in COVID-19 patients: A systematic review and meta-analysis
Source: PLoS One. 2022 Jul 28;17(7):e0271795. doi: 10.1371/journal.pone.0271795 (PMC9333218; doi:10.1371/journal.pone.0271795)
Supplement: S3 Table — (DOCX) [file pone.0271795.s006.docx]

**Supplementary Table S3: Risk of bias assessment for Case report and Case series using methodological quality and synthesis**

| **First author, Country, year** | **Type of literature** | **Domains** | | | | | **Total score** | **Levels of RoB** |
| --- | --- | --- | --- | --- | --- | --- | --- | --- |
|  |  | **Selection** | **Ascertainment** | | **Causality** | **Reporting** |  |  |
|  |  | **Q1** | **Q2** | **Q3** | **Q7** | **Q8** |  |  |
| Haglund A et al, Denmark, 2021 | Case report | 1 | 1 | 1 | 1 | 1 | 5 | Low |
| Hakamifard A et al, Iran, 2020 | Case report | 1 | 1 | 1 | 1 | 1 | 5 | Low |
| Sharma A et al, Australia, 2021 | Case report | 1 | 1 | 1 | 1 | 1 | 5 | Low |
| Witting C et al, USA, 2021 | Case report | 1 | 1 | 1 | 1 | 1 | 5 | Low |
| Deana C et al, Italy, 2021 | Case report | 1 | 1 | 1 | 1 | 1 | 5 | Low |
| Nasri E et al, Iran, 2020 | Case reprt | 1 | 1 | 1 | 1 | 1 | 5 | Low |
| Mohamed A et al, Ireland, 2021 | Case report | 1 | 1 | 1 | 1 | 1 | 5 | Low |
| Schein F et al. France, 2020 | Case report | 1 | 1 | 1 | 1 | 1 | 5 | Low |
| Trujillo H et al. Spain, 2020 | Case report | 1 | 1 | 1 | 1 | 1 | 5 | Low |
| Prattes J et al, USA, 2021 | Case report | 1 | 1 | 1 | 1 | 0 | 4 | Medium |
| Alobaid K et al, Kuwait, 2021 | Case reports | 1 | 1 | 1 | 1 | 1 | 5 | Low |
| Trovato L et al, Italy, 2020 | Case report | 1 | 1 | 1 | 1 | 1 | 5 | Low |
| Saccaro LF et al, Italy, 2020 | Case report | 1 | 1 | 1 | 1 | 1 | 5 | Low |
| Bilani N et al, USA, 2020 | Case report | 1 | 1 | 1 | 1 | 0 | 4 | Medium |
| Fernandez NB et al, Argentina, 2021 | Case report | 1 | 1 | 1 | 1 | 1 | 5 | Low |
| Patti RK et al, USA, 2020 | Case report | 1 | 1 | 1 | 1 | 1 | 5 | Low |
| Kakamad FH et al, Iraq, 2021 | Case report | 1 | 1 | 1 | 1 | 0 | 4 | Medium |
| Abdalla S et al, Qatar, 2020 | Case reports | 1 | 1 | 1 | 1 | 1 | 5 | Low |
| Imoto M et al, Japan, 2021 | Case report | 1 | 1 | 1 | 1 | 1 | 5 | Low |
| Iwanaga Y et al, Japan, 2021 | Case report | 1 | 1 | 1 | 1 | 0 | 4 | Medium |
| Maini A et al, India, 2021 | Case report | 1 | 1 | 1 | 1 | 1 | 5 | Low |
| Khatri A et al, USA, 2021 | Case report | 1 | 1 | 1 | 1 | 1 | 5 | Low |
| Arana C et al, Spain, 2021 | Case reports | 1 | 1 | 1 | 1 | 1 | 5 | Low |
| Krishna DS et al, India, 2021 | Case reports | 1 | 1 | 1 | 1 | 0 | 4 | Medium |
| Garg D et al, India, 2021 | Case report | 1 | 1 | 1 | 1 | 1 | 5 | Low |
| Junior ESM et al, Brazil, 2020 | Case report | 1 | 1 | 1 | 1 | 0 | 4 | Medium |
| Revannavar SM et al, India, 2021 | Case report | 1 | 1 | 1 | 1 | 0 | 4 | Medium |
| Sari AP et al, Indonesia, 2021 | Case report | 1 | 1 | 1 | 1 | 1 | 5 | Low |
| Chang CC et al, USA, 2020 | Case report | 1 | 1 | 1 | 1 | 0 | 4 | Medium |
| Ventoulis I et al, Greece, 2020 | Case reports | 1 | 1 | 1 | 1 | 0 | 4 | Medium |
| Bertolini M et al, Argentina, 2020 | Case report | 1 | 1 | 1 | 1 | 1 | 5 | Low |
| Khatib MY et al, Qatar, 2020 | Case report | 1 | 1 | 1 | 1 | 1 | 5 | Low |
| Seitz T eta al, Austria, 2020 | Case report | 1 | 1 | 1 | 1 | 1 | 5 | Low |
| Meijer EFJ et al, Netherlands, 2020 | Case series | 1 | 1 | 1 | 1 | 1 | 5 | Low |
| Benedetti MF (2021), Argentina | Case series | 1 | 1 | 1 | 1 | 1 | 5 | Low |
| Flikweert AW et al, Netherlands, 2020 | Case series | 1 | 1 | 1 | 1 | 1 | 5 | Low |
| Q1. Does the patient(s) represent(s) the whole experience of the investigator (centre) or is the selection method unclear to the extent that other patients with similar presentation may not have been reported? -Applicable  Q2. Was the exposure adequately ascertained? - Applicable  Q3. Was the outcome adequately ascertained? -Applicable  Q4. Were other alternative causes that may explain the observation ruled out? - Not applicable  Q5. Was there a challenge/rechallenge phenomenon? - Not applicable  Q6. Was there a dose–response effect? - Not applicable  Q7. Was follow-up long enough for outcomes to occur? - Applicable  Q8. Is the case(s) described with sufficient details to allow other investigators to replicate the research or to allow practitioners make inferences related to their own practice? -Applicable | | | | | | | | |
