# Supplementary material for: Antifungal therapy in the management of fungal secondary infections in COVID-19 patients: A systematic review and meta-analysis
Source: PLoS One. 2022 Jul 28;17(7):e0271795. doi: 10.1371/journal.pone.0271795 (PMC9333218; doi:10.1371/journal.pone.0271795)
Supplement: S4 Table — (DOCX) [file pone.0271795.s007.docx]

**Supplementary Table S4: Risk of bias assessment for Cohort studies using Newcastle-Ottawa Quality assessment**

| **Author, Country, Year of publication** | **Selection** | | | | **Comparability** | | **Outcome** | | | **Total quality score** | **Corresponding Quality** | **RoB** |
| --- | --- | --- | --- | --- | --- | --- | --- | --- | --- | --- | --- | --- |
|  | **1** | **2** | **3** | **4** | **1. a (age)** | **1. b** | **1** | **2** | **3** |  |  |  |
| Rothe K et al, Germany, 2021 | * | * | * | 0 | * | 0 | * | * | 0 | 6 | Good | Moderate |
| Sen M et al, India, 2021 | * | * | * | 0 | * | 0 | * | * | * | 7 | Good | Low |
| Chen N et al, China, 2020 | * | * | * | 0 | * | 0 | * | * | * | 7 | Good | Low |
| Permpalung N et al, USA, 2021 | * | * | * | 0 | * | 0 | * | * | 0 | 6 | Good | Moderate |
| White PL et al, UK, 2020 | * | * | * | 0 | * | 0 | * | * | * | 7 | Good | Low |
| Koehler P et al, Germany, 2020 | * | * | * | 0 | * | 0 | * | 0 | * | 6 | Good | Moderate |
| Maes M et al, UK, 2021 | * | 0 | * | 0 | * | 0 | * | 0 | * | 5 | Good | Moderate |
| Nasir N et al, Pakistan, 2020 | * | * | * | 0 | * | 0 | * | * | * | 7 | Good | Low |
| Bishburg E et al, Israel, USA, 2020 | * | * | * | 0 | * | 0 | * | * | 0 | 6 | Good | Moderate |
| Lahmer T et al, Germany, 2021 | * | * | * | 0 | * | 0 | * | * | 0 | 6 | Good | Moderate |
| Arastehfar A et al, Iran, 2021 | * | * | * | 0 | * | 0 | * | * | * | 7 | Good | Low |
| Fekkar A et al, France, 2021 | * | * | * | 0 | * | 0 | * | * | 0 | 6 | Good | Moderate |
| Roman-Montes CM et al, Mexico, 2020 | * | * | * | 0 | * | 0 | * | * | * | 7 | Good | Low |
| Segrelles-Calvo G et al, Spain, 2020 | * | * | * | 0 | * | 0 | * | * | * | 7 | Good | Low |
| Mitaka H et al, USA, 2020 | * | * | * | 0 | * | 0 | * | * | 0 | 6 | Good | Moderate |
| Salmanton-García J et al, Germany, 2021 | * | * | * | 0 | * | 0 | * | * | * | 7 | Good | Low |
| Søgaard KK et al, Switzerland, 2021 | * | * | * | 0 | * | 0 | * | 0 | 0 | 5 | Good | Moderate |
| Versyck M et al, France, 2021 | * | * | * | 0 | * | 0 | * | * | * | 7 | Good | Low |
| Salehi M et al, Iran, 2020 | * | * | * | 0 | * | 0 | * | 0 | 0 | 5 | Good | Moderate |
| Buehler PK et al, Switzerland, 2020 | * | * | * | 0 | * | 0 | * | 0 | 0 | 5 | Good | Moderate |
| Seaton RA et al, UK, 2020 | * | * | * | 0 | * | 0 | * | 0 | 0 | 5 | Good | Moderate |

Total score of three or less was regarded as indicative of poor quality; 4–6 was regarded as moderate quality; 7–9 was regarded as high quality
