# Supplementary material for: Antifungal therapy in the management of fungal secondary infections in COVID-19 patients: A systematic review and meta-analysis
Source: PLoS One. 2022 Jul 28;17(7):e0271795. doi: 10.1371/journal.pone.0271795 (PMC9333218; doi:10.1371/journal.pone.0271795)
Supplement: S5 Table — (DOCX) [file pone.0271795.s008.docx]

**Supplementary Table S5: Details of ongoing randomized control studies involving COVID-19 patients with fungal secondary infections**

| **Study identifier and phase of study** | **Title** | **Intervention group** | **Control group** | **Expected outcomes** |
| --- | --- | --- | --- | --- |
| NCT04707703 / Phase 3 | Isavuconazole for the Prevention of COVID-19-associated Pulmonary Aspergillosis (Isavu-CAPA) | IV Isavuconazonium sulfate 372 mg every 8 hours for 6 doses, followed by 372 mg once daily for 28 days | Placebo | - Incidence of invasive aspergillosis at time of ICU discharge - Incidence of non-invasive aspergillosis at time of ICU discharge - Survival rate - Length of ICU stay - Length of hospital stay - Mortality - Adverse events |
| NCT04577378 / Phase 2 | Efficacy and Safety of Drug Combination Therapy of Isotretinoin and Some Antifungal Drugs as A Potential Aerosol Therapy for COVID-19 : An Innovative Therapeutic Approach COVID-19 (Isotretinoin) | - Isotretinoin(Aerosolized 13 cis retinoic acid) plus Aerosolized Itraconazole: Aerosolized 13 cis retinoic acid in gradual one dose increases from 0.2 to 4 mg/kg/day as inhaled 13 cis retinoic acid therapy plus Aerosolized Itraconazole 5mg per day for 14 days - Aerosolized 13 cis retinoic acid in gradual one dose increases from 0.2 to 4 mg/kg/day as inhaled 13 cis retinoic acid therapy for 14 days | 13 cis retinoic acid doses orally | - Lung injury score - Absolute lymphocyte counts - Serum levels of CRP, ESR ,IL-1,IL-6,TNF and Type I interferons - All cause mortality rate - Serum level of viral RNA - Ventilation free days - ICU free days |
