# Supplementary material for: Antifungal therapy in the management of fungal secondary infections in COVID-19 patients: A systematic review and meta-analysis
Source: PLoS One. 2022 Jul 28;17(7):e0271795. doi: 10.1371/journal.pone.0271795 (PMC9333218; doi:10.1371/journal.pone.0271795)
Supplement: Attachment — Submitted filename: Response to reviewer.docx [file pone.0271795.s009.docx]

| **Manuscript ID:** | | PONE-D-21-30319 | | |
| --- | --- | --- | --- | --- |
| **Manuscript title:** | | Antifungal therapy in the management of fungal co-infections in COVID-19 patients: A systematic review and meta-analysis | | |
| **Revision 1** | | | | |
| **Reviwer-1** | | | | |
| **Sl. No** | **Reviewer comments** | | **Responses/Rebuttal** | **Corresponding changes in the revised manuscript** |
| 1. | The funding statement is not clear, was funding received for this manuscript and if so, from which entity? | | Attended | The funding details have been updated in the revised manuscript and **highlighted**. [Page no. 18; line number: 413-414] |
| 2. | It should be made clear in the abstract and earlier in the methods that these study includes an individual patient meta-analysis. Perhaps it is also necessary to include criteria for inclusion into the SR vs. into the IPD meta-analysis, as these appear to be slightly different populations in this study. For example, did studies have to have IPD in order to be included in the SR? Perhaps PRISMA-IPD checklist should be used for this study? | | **Attended**  **Response to the author:** We appreciate your suggestion for looking to IPD meta-analysis, which was new to us. We went through various related articles and found that IPD meta-analysis is also used to retrieve unpublished information from clinical studies (especially clinical trials comparing interventions). However, in our study, different populations and study designs were used but we could not able to find any clinical trial-related studies during data extraction. We included all published data except one study (reference number 77). For that one study, we contacted the original study author and gathered information. Soon after that with the same information that article got published.  In this review, we tried to compare the outcomes within the cohort studies which may create scope for the IPD meta-analysis. With concern that as well as reviewer suggestions we contacted selected articles’ authors through the mail but their response was very poor. To date, they were not provided information as the information was published in cohort studies.  Due to these difficulties in this review, we were unable to include IPD meta-analysis but added a few critical points in the conclusion that may be helpful to resolve this problem. | - |
| 3. | Please provide a justification for why only English language studies were searched given the global impact of COVID-19 and potential squeal. | | Attended  **Response to the reviewer:** The main reason behind selecting only the English language was that English is most commonly readable, understandable and easily reachable to international researchers. Further, selecting multiple language articles may require personnel who were multiple language experts, which is difficult to get. Therefore, we selected the standard language English, which is spoken and written by the majority of researchers around the globe. | - |
| 4. | Minor: please correct spelling of clinicaltrial.gov | | Attended & Corrected | Spelling of **ClinicalTrial.gov** has been corrected in the method section of the abstract [page no. 3; line number: 69] and in the main manuscript [page no. 6; line number: 146], same has been **highlighted** in the revised manuscript. |
| 5. | Please clarify whether full text screening was performed in duplicate. | | Attended  **Response to reviewer:** The identified records’ title were screened during duplication removal. However, for the study of relevant publications, both title and full text were reviewed before removing duplicates. | - |
| 6. | How was missing data handled? Presumably some studies will report prevalence of anti-fungal use but not on patient outcomes. | | **Attended**  **Response to reviewer:** If any study relevant data was missing or incomplete then review authors were engaged to search original sources or contacted original authors through mail to obtain any missing information. If it is not possible to obtain missing information, then those studies were excluded from this review. | The details have been updated in the study **selection section** in methods and **highlighted** in the revised manuscript. [page no. 7; line number: 158-160] |
| 7. | Are the authors able to stratify patients by those with fungal co-infections on admission vs. later in hospital stay (e.g. > 48h)? This could be helpful to delineate outcomes between "co-infections" and "secondary or nosocomial infections". | | Attended:  **Response to Reviewer:** Initially we attend to stratify patients by those with fungal co-infections on admission vs later in hospital stay but we found major issues with many patients that they had more than one hospital admission and delay in detection of antifungal co-infections. Most of the time patients developed multiple coinfections together and made difficulties in obtaining the exact time of fungal infections. These made it difficult in obtaining exact information. | - |
| 8. | The search yield seems extremely low given the volume of studies on COVID-19 up to present. Can the authors explain why they applied "Review, Systematic review" filters in pubmed if they were not aiming to include these studies. The pubmed filters seem to narrow the results significantly. | | Attended:  **Response to Reviewer:** During PubMed's initial search we included Review and Systematic review terms search to extract the maximum volume of studies. We included all the fields during the search in PubMed and inadvertently mentioned them in a confusing way. Clarity has been provided and updated in the revised manuscript. | Corrections have been made and uploaded in Supplementary Appendix S2 and highlighted. [Supplementary file: Page number 5] |
| 9. | Please indicate a measure of patient severity, e.g., how many in ICU or on mechanical ventilation? | | Attended  **Response to reviewer:** Most of the patients from included studies were admitted to ICU or received mechanical ventilation during their hospital stay. As per the reviewer's suggestion, we authors attempted to address this and updated in table 1 and table 2. | The details are updated in the result section [page no. 11; line number: 262-263] and in table 1 and table 2. The same has been **highlighted** in the revised manuscript. |
| 10. | Were any studies (cohort) included that exclusively looked at patients with fungal infections? The rate of fungal infections seems high and could be elevated based on the denominator selected. | | Attended  **Response to reviewer:** The selected cohort study were included patients with fungal infections and evaluated outcomes in various domains such as identifying prevalence, the effectiveness of the diagnostic test, duration of hospital stay, ICU admission, clinical features, diagnostic tests, mortality, co-infections and their management. However, exactly no studies were evaluated the efficacy of antifungal agents in fungal co-infection patients with COVID-19. We rigorously analysed the data from each study and draw the correct information. | - |
| 11. | Can the authors provide additional detail on the microbiological methods used for detection of fungi, as well as the clinical criteria used to identify true infections? | | Attended | The details have been updated in the methods section and **highlighted** in the revised manuscript. [page no. 6; line number: 136-137] |
| 12. | How many patients were eligible for IPD meta-analysis? The numbers in forest plots seem very low and not conducive to drawing conclusions on outcomes in this patient population. | | **Attended**  **Response to reviewer:** Please look for the responses in query 2. However, we added a few critical points in conclusion to resolve this issue. | The details have been uploaded in the conclusion section of the abstract [page no. 4; line number: 83, 85 and 87-89] and in the main manuscript. [page no. 17; line number: 407-409] |
| 13. | Comparing all-cause mortality among type and duration of AFT doesn't seem appropriate given 1) the low sample size and 2) the lack of accounting for confounding factors. | | Attended | The details have been updated and highlighted in the revised manuscript. [page no. 15-16; line number: 362-367] |
| **Reviewer-2** | | | | |
| 1 | A critical point is the use of “unpublished material”. This kind of data is not easily available to anyone. So, what is the rational for using material that has not been peer reviewed and accessible to any researcher? Unless is from bioRxiv, for example. This needs to be very well clarified. | | Attended  **Response to the reviewer:** We authors were aware that data from unpublished studies can itself introduce bias to the study. However, the current study was emerging and multiple publications were published on pre-print websites before getting peer-reviewed. This publication may have study-related important information, which can be obtained by contacting the original authors.    In the current study, we tried to gather data from all types of possible databases to strengthen the search strategies. However, the record which we identified from unpublished databases were excluded during screening itself due to inclusion criteria except one (reference number 77). For that one study, we contacted the original study author and gathered information. Soon after that with the same information that article got published.  .https://doi.org/10.1016/j.xcrm.2021.100229 | The details about how we gathered missing information have been updated and **highlighted** in the revised manuscript. [page no. 7; line number: 158-160] |
| 2. | Introduction  - “The exact incidence of fungal co-infections in COVID-19 patients has not been established.” – this reviewer does not agree with this sentence. Please check these works, where this has been estimated (in general or locally): doi: 10.1007/s11046-020-00462-9; DOI: 10.3390/jof7090720; doi: 10.1016/j.cmi.2020.06.025 and correct the information;  - In terms of drugs, I would recommend checking this recent work: doi: 10.1016/j.jmii.2020.05.013; | | Attended  **Response to reviewer:** This issue was reviewed carefully and corrected. | The sentence has been re-structured after correction. The details have been **highlighted** in the revised manuscript. [page no. 5; line number: 109-111]  **Reference:** Information was taken from reference number 2.  Song G, Liang G, Liu W. Fungal Co-infections Associated with Global COVID-19 Pandemic: A Clinical and Diagnostic Perspective from China. Mycopathologia. 2020;185(4): 599–606. doi:10.1007/s11046-020-00462-9 |
| 3 | - “The commonly used antifungal therapy (AFT) include liposomal amphotericin B, azole antifungals,and echinocandins[6].” – antifungals after “azoles” is unnecessary. All examples are antifungals. Correct this in the entire MS; | | Attended  **Response to reviewer:** This issue was corrected throughout the manuscript**.** | As per the reviewer's suggestion, the word **antifungal** after azole has been removed throughout the manuscript in the revised manuscript.   a. [Page number 5, line number 123] b. [Page number 10, line number 246] c. [Page number 11, line number 249] d. [Page number 11, line number 252] e. [Page number 15, line number 350] |
| 4 | M&M: RevMan 5.4 – country and manufacturer? | | Attended | The details have been updated and **highlighted** in the revised manuscript in the statistical analysis section of methods. [page no. 8; line number: 184-186] |
| 5 | Results:  - This section is too long. The MS provides nice tables, so some of the information should be summarized. Is too exhaustive. | | Attended  **Response to reviewer:** We presented results in various possible ways. However, due to the reviewer's suggestion, few sentences were re-structured to reduce the length of the result section without altering the main findings. | The sentences have been re-structured and **highlighted** in the revised manuscript.  [page no. 11; line number: 260]  [page no. 12; line number: 271-172]  [page no. 12; line number: 277-278] |
| **6.** | Grammar, spelling and punctuation have been corrected throughout the manuscript and **highlighted** in the revised manuscript | | | |
| **Revsison 2 (Editor comments and response)** | | | | |
| **1.** | We notice that your manuscript file was uploaded on Sep 19 2021. Please can you upload the latest version of your revised manuscript as the main article file. | | Attended | Changes has been made and uploaded revised manuscript without tracker changes. |
| **2.** | Please include your amended statements within your cover letter. | | Attended | Included in cover letter and uploaded |
| **3.** | PLOS requires an ORCID iD for the corresponding author | | Attended | Added ORCID ID of corresponding author |
| **4.** | Your article cannot proceed until you upload a copy of the completed PRISMA checklist as Supporting Information. We note that this manuscript is a systematic review or meta-analysis; our author guidelines therefore require that you use PRISMA guidance to help improve reporting quality of this type of study. | | Attended | Prisma check list has been uploaded along with the revised supporting file. [Page number: 2-3] |
