# Supplementary material for: Antifungal therapy in the management of fungal secondary infections in COVID-19 patients: A systematic review and meta-analysis
Source: PLoS One. 2022 Jul 28;17(7):e0271795. doi: 10.1371/journal.pone.0271795 (PMC9333218; doi:10.1371/journal.pone.0271795)
Supplement: Attachment — Submitted filename: Response to reviewer.docx [file pone.0271795.s010.docx]

| **Manuscript ID:** | | PONE-D-21-30319 | | |
| --- | --- | --- | --- | --- |
| **Manuscript title:** | | Antifungal therapy in the management of fungal co-infections in COVID-19 patients: A systematic review and meta-analysis | | |
| **Revision 2** | | | | |
| **Reviwer-1** | | | | |
| **Sl. No** | **Reviewer comments** | | **Responses/Rebuttal** | **Corresponding changes in the revised manuscript** |
| 1. | Consider the term "fungal co-infection or secondary infection" rather than "co-infection" since they cannot be differentiated/disaggregated in this study. Co-infection generally refers to upon presentation or <48h after presentation with COVID-19, and it would be unlikely that all patients with fungal co-infection developed early after presentation. | | Attended  **Response to the reviewer:** We appreciate your suggestion and found using “fungal secondary infections” is suitable medical term instead of “fungal co-infections” in this study and the same has been changed throughout the manuscript. | The changes have been updated in the revised manuscript and **highlighted** in the title section, abstract, Introduction, Methods, Results, Discussion, Conclusion, Figure and supplementary files. |
| 2. | Please clarify if any, and how many, studies included patients with suspected but not confirmed COVID-19. The methods indicate that patients must have had confirmed COVID-19, but for example Seaton et al, included many patients without positive SARS-CoV-2 tests. 10.1016/j.jinf.2020.09.024 | | **Attended**  **Response to the reviewer:** In this study, we only included the 479 patients out of 1537 COVID 19 patients, who was confirmed case of COVID-19 and diagnosed with fungal secondary infections. Hence, we excluded all the patients who were not confirmed cases of COVID-19. Majority of selected Cohort studies included patients with suspected but not confirmed COVID-19. | The details have been previously addressed in the result section “3.4. Participants’ characteristics and clinical diagnosis”. [Page 10, line number: 231-233] |
| 3. | The Seaton study is referenced by the authors as occurring in UK and Germany, but the study itself only indicates UK. | | **Attended**  **Response to the reviewer:** Even though the authors are from various countries, this study was only conducted in the UK. Inadvertently added in result section in Study characteristics. | The issue has been corrected and highlighted in the main manuscript in the revised manuscript. [Page 9, line number: 213] |
| 4. | Thank you for adding a statement on limitations line 407-409, but this should be in limitations rather than conclusions. Line 408-409, please rephrase as a small number of events is not a confounding factor, but rather leads to reduced power to detect a difference when in fact one may exist. | | **Attended** | The sentence has been rephrased and highlighted in the revised manuscript.  [Page 17, line number: 412-413] |
